# Occurrence, concentration, and risk assessment of selected pharmaceuticals in representative cropland soils and their underlying groundwater in Gauteng province, South Africa

**DOI:** 10.1007/s10661-025-14436-1

**Published:** 2025-08-06

**Authors:** Matome Peter Ngoetjana, Eyob Habte Tesfamariam, Sally Brown, Madelien Wooding, Matthys Alois Dippenaar

**Affiliations:** 1 Department of Plant and Soil Science, Faculty of Natural and Agricultural Science, University of Pretoria, Pretoria, South Africa; 2 School of Environmental and Forest Sciences, University of Washington, Seattle, WA USA; 3 Department of Chemistry, Faculty of Natural and Agricultural Science, University of Pretoria, Pretoria, South Africa; 4 Department of Geology, Faculty of Natural and Agricultural Science, University of Pretoria, Pretoria, South Africa

**Keywords:** Occurrence, Risk, Pharmaceuticals, Cropland, Groundwater

## Abstract

Traceable levels of pharmaceuticals are frequently detected in various environmental matrices. However, studies investigating the occurrence of pharmaceuticals in cropland soils and underlying groundwaters are relatively limited, hindering risk assessments. This study aims the following: (1) determine the occurrence of selected pharmaceuticals in cropland soils and underlying groundwater, (2) investigate the correlations between pharmaceutical’s occurrence in cropland soils and underlying groundwater, (3) evaluate the correlations between soil and water parameters and pharmaceutical’s concentration, and (4) estimate human health risk from pharmaceuticals present in groundwater. A single soil and groundwater sampling campaign was conducted across eighteen farmers’ fields, characterized by varying farm management practices. Caffeine, carbamazepine-10,11-epoxide, carbamazepine diol, and N4-acetylsulfamethoxazole were detected in groundwater, with concentrations reaching 67.1, 106.7, 506.7, and 113.8 ng L^−1^, respectively. Three of these compounds, carbamazepine-10,11-epoxide, carbamazepine diol, and N4-acetylsulfamethoxazole, were also present in soils with concentrations reaching 10.0, 353.5, and 59.1 ng g^−1^ dry weight, respectively, indicating potential soil-to-groundwater transfer. Principal component analysis revealed a strong correlation between the application of commercial inorganic fertilizers and carbamazepine-10,11-epoxide levels and the irrigation practices and the levels of caffeine, carbamazepine diol, and N4-acetylsulfamethoxazole in the soil and groundwater. Risk quotients showed that caffeine poses negligible risk to human health through drinking water exposure. Overall, the findings suggest that pharmaceuticals are present in South African croplands and underlying groundwater, though current levels do not pose a significant risk to human health. However, future studies should focus on other metabolites of pharmaceuticals due to their higher concentrations in the soil and groundwater.

**Supplementary Information:**

The online version contains supplementary material available at 10.1007/s10661-025-14436-1.

## Introduction

Groundwater remains one of the most reliable sources of direct potable water in South Africa and worldwide. According to the Water Research Commission, more than 50% of South Africa’s rural population relies on groundwater as a source of potable water (WRC, 2017). In Europe, groundwater contributes more than 60% of potable water usage (EEA, 2022). In Asia, more than 70% of the population relies on groundwater as a source of potable water (Carrard et al., 2019). With such high reliance on groundwater in most parts of the world, there is a need to protect our groundwater resources from all sorts of contamination, including pharmaceuticals that are currently threatening the state of the water quality across the globe.

Soils are recognized as a significant pathway for the entry of pharmaceuticals into groundwater through leaching (Silori et al., 2022). This phenomenon is particularly pronounced in regions where there is a direct connection between the vadose zone and groundwater. Several studies have documented the presence of pharmaceuticals in agricultural lands that have been amended with wastewater (Biel-Maeso et al., 2018; Liu et al., 2020), municipal biosolids (Bastos et al., 2020; Martín et al., 2021), and animal manure (Conde-Cid et al., 2018; Qian et al., 2022). Similarly, several other studies have detected pharmaceuticals in groundwater near surface water bodies (Ma et al., 2022; Yang et al., 2017) at points where wastewater treatment effluent is discharged (Arun et al., 2022; Bolujoko et al., 2024), and in proximity to landfill sites (Han et al., 2022; Wang et al., 2021). However, there is a dearth of studies examining the presence of pharmaceuticals in groundwater beneath croplands. To our knowledge, no studies have explored the relationship between fertilization practices and supplemental irrigation on the concentration of pharmaceuticals in groundwater. Moreover, research on the correlation between soil properties and pharmaceuticals concentrations in soils is also quite limited, if it exists at all.

In South Africa, the occurrence of pharmaceuticals is also inadequately monitored in cropland soils and their underlying groundwater. Most studies focus on the occurrence of pharmaceuticals in treated municipal wastewater and surface water (Gani et al., 2021). Currently, there is no information on the status of pharmaceuticals in South African cropland soils. Furthermore, only a few studies in the country focused on pharmaceuticals occurrence in groundwater (Rimayi et al., 2018). The study by Rimayi et al. (2018) monitored the occurrence of pharmaceutical compounds, including five antiretroviral drugs (nevirapine, efavirenz, lamivudine, emtricitabine, and tenofovir disoproxil) and an anticonvulsant (carbamazepine) in groundwater near the Hartbeespoort Dam, Gauteng province. Nevirapine, efavirenz, and carbamazepine were detected in groundwater at concentrations up to 13, 5, and 13 ng L^−1^, respectively (Rimayi et al., 2018). However, the study by Rimayi et al. (2018) monitored pharmaceuticals in groundwater near a surface water body and is not representative of groundwater underlying agricultural lands due to various factors such as differences in water sources, recharge rates, and aquifer characteristics.

This study aimed the following: (1) investigate the occurrence of selected pharmaceuticals in cropland soils and underlying groundwater in Gauteng province, South Africa; (2) investigate the correlations between pharmaceutical’s occurrence in cropland soils and underlying groundwater; (3) evaluate the correlations between soil and water physicochemical parameters and pharmaceutical’s concentration in both cropland soil and underlying groundwater, respectively; (4) evaluate the correlations between farm management practices and pharmaceutical’s occurrence in groundwater and cropland soils; and (5) estimate the human health risk associated with detected pharmaceuticals in groundwater. We hypothesized that: (1) pharmaceuticals contamination occurs in cropland soils and underlying groundwater, irrespective of farm management practice; (2) soil and groundwater pharmaceutical’s occurrence are positively correlated, (3) soil pharmaceutical levels are positively correlated with soil organic matter and clay content, while groundwater pharmaceuticals levels show a negative correlation; (4) irrigation and biowaste-based fertilization increase pharmaceuticals occurrence and concentration in both soil and groundwater; and (5) current pharmaceuticals concentrations in groundwater pose minimal risk to human health.

## Material and methods

### General description of the study area

#### Location and climate

**Fig. 1:**
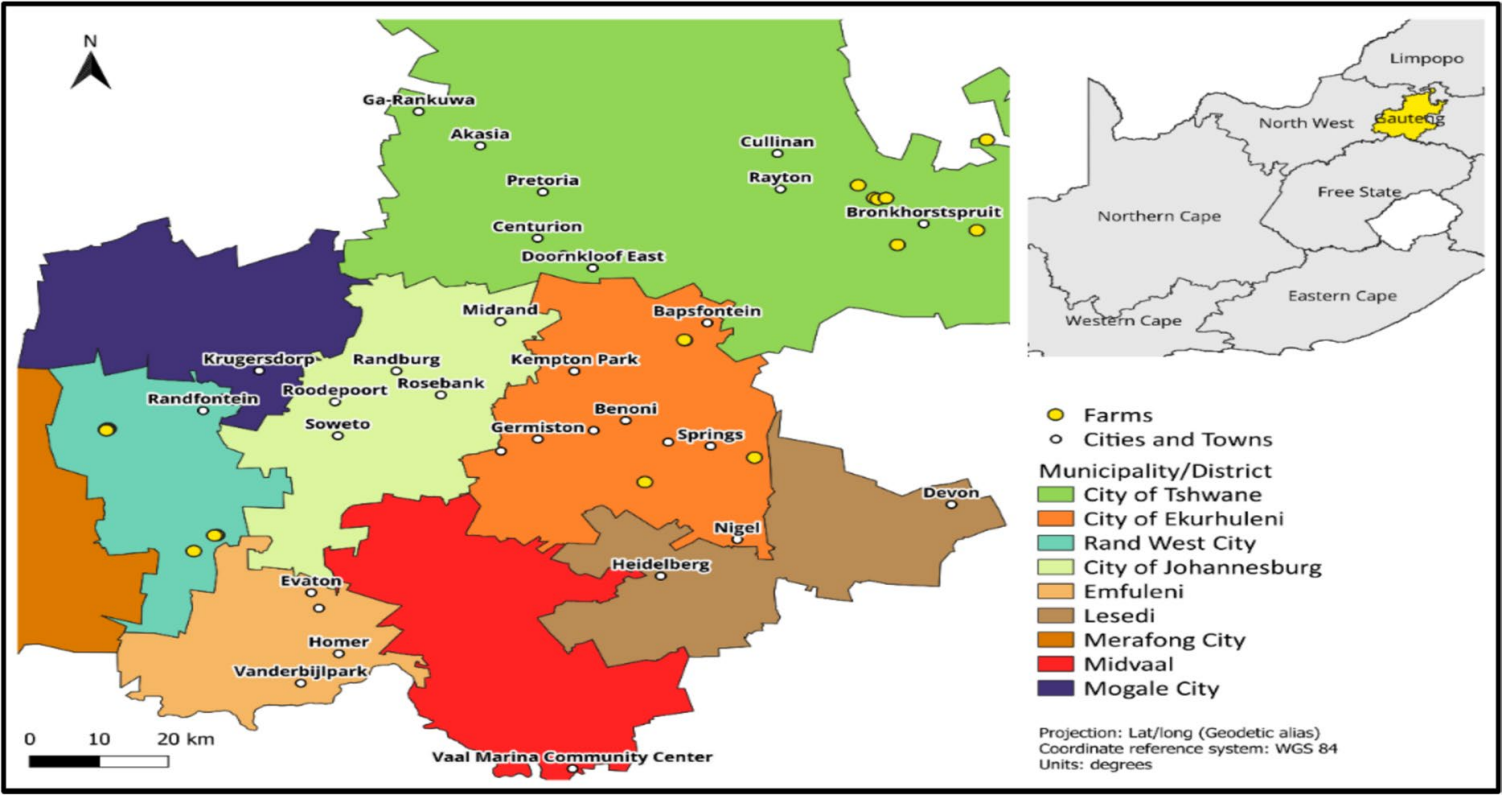
The map of South Africa (top right) showing the Gauteng province (left) and the location of the farms (yellow) where the study was conducted

The study was carried out in croplands across three district municipalities: Tshwane, Ekurhuleni, and Westrand, situated in Gauteng province, South Africa (Fig. 1). Gauteng, known as South Africa’s economic hub, is a highly industrialized region with a population density surpassing that of other provinces (StatsSA, 2022). The province lies within a sub-humid zone, experiencing annual summer rainfall ranging from 600 to 800 mm, predominantly from October to March (Symes et al., 2017).

#### Soil and land use

The soils in Gauteng are predominantly sandy clay loam and sandy loam (Land Type Survey Staff, 2006), characterized by low productivity due to low organic matter (≤ 2%) (Stronkhorst & Venter, 2008) and acidic pH levels (≤ 6.4) (ARC–ISCW, 2006). Consequently, farmers frequently apply high doses of commercial inorganic fertilizers to meet the crop nutrient demands, while the use of animal manure is rare. The main crops cultivated in the province include vegetables, maize, beans, and sunflowers (Nesamvuni et al., 2016).

#### Hydrogeology

From the hydrogeological point of view, the study sites are characterized by shallow to intermediate semi-confined aquifers, with general depth to groundwater ranging from 50 to 100 m. The aquifers are predominantly overlain by the shale and sandstone formation of the Karoo Supergroup (Johnson et al., 2006). Sandstone units act as primary aquifers and are characterized by moderate to high permeability and porosity (Todd & Mays, 2004). Shale units act as aquitards, restricting the downward movement of water and confining groundwater within deeper sandstone layers (Todd & Mays, 2004). Aquifers are mainly recharged by precipitation and the process occurs in exposed sandstone outcrops (Stuart & Smith, 2016). Surface water bodies and irrigation water serve as secondary recharge sources mainly through seepage and fractured rocks (Stuart & Smith, 2016). Hence, groundwater levels vary seasonally and are expected to be low between May and September and high from October to March of the next year. While the sandstone-shale aquifers are semi-confined, their vulnerability to contamination can be influenced by the pollution taking place in the vadose zone.

### Selection of sampling sites

Eighteen croplands (*n* = 18) were selected for soil and groundwater sampling from the three district municipalities in Gauteng province, South Africa. These croplands were included in the current study based on specific selection criteria: (1) availability of boreholes, (2) use of most common fertilization systems in South Africa, commercial inorganic fertilizers and/or animal manure, (3) implementation of water management practices that encompass rainfed and/or irrigated systems, and (4) cultivation of common food crops in South Africa such as vegetables, legumes, and/or cereals. Table 1 presents an overview of key soil parameters, water management practices, fertilization systems, and the type of food crops found on the eighteen croplands included in this investigation.

**Table 1:** Geographical coordinates, soil parameters, water managements, fertilization systems, and farming practices of the farmers’ fields under investigation

Farm	Latitude	Longitude	FP	WM	FS	pH	%Sand	%Clay	%OC
A	S 25° 43′ 57.7″	E 028° 38′ 45.8″	Vegetable	Irrigated	CIF + AM	4.29	83	10	0.66
B	S 25° 49′ 14.8″	E 028° 49′ 07.0″	Vegetable	Irrigated	CIF	6.7	82	10	0.91
C	S 25° 45′ 27.6″	E 028° 40′ 11.0″	Cereal	Rainfed	CIF	4.85	87	8	0.74
D	S 25° 45′ 36.6″	E 028° 40′ 29.4″	Legume	Rainfed	CIF	4.85	88	8	0.74
E	S 25° 45′ 28.0″	E 028° 41′ 11.8″	Cereal	Rainfed	CIF + AM	5.54	81	10	0.66
F	S 25° 38′ 37.5″	E 028° 49′ 58.5″	Cereal	Rainfed	CIF + AM	4.43	60	24	0.91
G	S 26° 02′ 09.3″	E 028° 23′ 38.7″	Vegetable	Irrigated	CIF + AM	5.11	68	30	0.66
H	S 26° 02′ 06.4″	E 028° 23′ 36.7″	Vegetable	Irrigated	CIF	5.44	59	24	0.91
I	S 26° 15′ 55.9″	E 028° 29′ 43.8″	Vegetable	Irrigated	CIF + AM	4.87	48	30	1.07
J	S 26° 18′ 48.6″	E 028° 20′ 10.3″	Vegetable	Irrigated	CIF	5.03	47	30	0.66
K	S 26° 26′ 54.8″	E 027° 40′ 50.6″	Cereal	Rainfed	CIF	3.77	78	14	0.91
L	S 26° 25′ 01.7″	E 027° 42′ 51.0″	Cereal	Rainfed	CIF	4.09	78	12	0.66
M	S 26° 25′ 03.3″	E 027° 42′ 50.7″	Cereal	Rainfed	CIF	4.51	64	20	0.82
N	S 26° 25′ 04.3″	E 027° 42′ 38.4″	Legume	Rainfed	CIF	4.51	76	16	0.49
O	S 26° 12′ 35.5″	E 027° 33′ 12.9″	Cereal	Rainfed	CIF	5.71	79	12	0.41
P	S 26° 12′ 30.9″	E 027° 33′ 20.9″	Cereal	Rainfed	CIF	5.77	84	10	0.58
Q	S 26° 12′ 39.5″	E 027° 33′ 19.6″	Cereal	Rainfed	CIF	6.61	80	10	0.99
R	S 26° 12′ 43.6″	E 027° 33′ 12.9″	Cereal	Rainfed	CIF	4.18	82	12	0.33

*CIF* means commercial inorganic fertilisers, *AM* means animal manure, *OC* means organic carbon, *FP* means farming practice, *WM* means water management, and *FS* means fertilization system

### Sample collection, preparation, and storage

#### Soil

Before crop planting in September 2022, soil samples were collected from eighteen representative cropland sites, as well as non-cropland sites adjacent to the cropland sites with no history of farming. Soil samples were collected following a simple random sampling from the top 20 cm layer, where pharmaceuticals pollution is generally higher (Biel-Maeso et al., 2018). To avoid cross-contamination, the auger was washed with water and rinsed with deionized water and methanol after every sampling layer. At least 10 subsamples were collected from each cropland site as recommended by the US EPA (2002) and mixed to prepare composite representative samples. The composite samples were divided into two parts: one for analyzing physicochemical parameters and the other for analyzing pharmaceuticals. The second set of samples was transferred to aluminum foils and kept in a CAMP MASTER electric cooler box (Model CB46) for transportation to the laboratory. The samples for physicochemical parameters analysis were transferred inside zip-lock plastic bags and also transported to the laboratory. All soil samples were air-dried under room temperature, sieved with a 2 mm diameter sieve, and stored at − 20 °C until extraction.

#### Groundwater

After soil sampling, groundwater samples were collected using SinkFastTM polyethylene bailers from open boreholes and water pumps for fitted boreholes from the eighteen croplands where soil samples were also collected. The water samples were transferred into 2.5 L amber bottles and transported in an electric cooler box (Model CB46). Upon arrival at the laboratory, the samples were stored at 4 °C until extraction.

### Selection of target pharmaceuticals

The current study examined eight pharmaceuticals frequently detected in cropland soils and groundwater (Biel-Maeso et al., 2018; Saha et al., 2022). Some of these contaminants are also included in the list of priority emerging contaminants of concern for assessing water quality related to direct potable reuse in South Africa (Swartz et al., 2018). The compounds investigated comprised of sulfamethoxazole (SMX), diclofenac (DCF), caffeine (CAF), carbamazepine (CBZ), sulfamethoxazole-N1-glucuronide (SMX-N1-Glu), N4-acetyl sulfamethoxazole (Ac-SMX), carbamazepine-10,11-epoxide (CBZ-EP), and carbamazepine diol (CBZ-DiOH). The physicochemical parameters for the targeted contaminants are presented in the Supplementary Table S1.

### Sample extraction and clean up

#### Soil

Soil electrical conductivity (EC) and pH (H_2_O) were tested in a soil: water solution ratio of 1:2.5 using Consort EC (C861 model) and pH (C830 model) meters, respectively (McLean, 1982). Percentage soil organic carbon (%OC) was measured following Walkley–Black’s method (Walkley & Black, 1934). Particle size distribution was measured following the hydrometer technique (Bouyoucos, 1962).

All target pharmaceuticals were extracted simultaneously based on the approach developed by de Santiago-Martin et al. (2020). The process involved three sequential extractions. The first extraction involved adding 20 mL of acetonitrile to the spiked sample and sonicating for 30 min. The second extraction involved adding 15 mL of phosphate buffer solution (pH 2) to the previously extracted sample and vortexed to suspend solids. The final extraction involved adding 15 mL of acetonitrile to the previously extracted sample and sonicating for 30 min. After each extraction, the mixture was centrifuged at a speed of 3000 rpm for 5 min, and the aqueous was removed into a clean test tube. The extracts from the three sequential extractions were combined inside a Buchner flask, and the solvents were evaporated using a rotary evaporator to a volume of about 20 mL. The extracts were then transferred to a 100 mL volumetric flask, and 250 mg Na_2_EDTA.2H_2_O was added. After adding Na_2_EDTA.2H_2_O, the volume was filled to a 100 mL mark with ultrapure water. The sample was loaded into the Oasis HLB 6 cc (6 mL, 200 mg, waters) cartridges, conditioned sequentially with 6 mL methanol, 6 mL ultrapure water, and 6 mL acidified ultrapure water (pH = 2). After loading the spiked sample, interfering materials were washed with 20 mL ultrapure water. The cartridges were then dried under vacuum (at 5 bar pressure) for 5 min. Target analytes were eluted using 3 × 4 mL aliquots of methanol, which was then evaporated using GeneVac (EZ-2 Plus series) at a heating temperature of 45 °C and a pressure of ≤ 200 mbar. Finally, analytes were recovered with 900 µL water: methanol (50:50, v/v) and 100 µL of a of a 1000 ng mL^−1^ isotope-labeled internal standard mixture of sulfamethoxazole and caffeine. The mixture was vortexed for 1 min, transferred to a 2 mL amber glass vial, and the samples were analyzed using Ultra Performance Liquid Chromatography with Quadrupole Time-of-Flight Mass Spectrometry (UPLC-QTOF).

#### Groundwater

Onsite measurements of the water samples’ physicochemical parameters, including pH, electrical conductivity, dissolved oxygen, and total dissolved solids, were conducted using the Lovibond multiparameter water sensor (SensoDirect 150).

The EPA method 1694, with certain modifications based on the approach developed by de Santiago-Martin et al. (2020), was utilized to extract pharmaceuticals from water samples. Water samples were filtered through a 0.7 µm glass fiber filter to remove solid residues. Subsequently, a 500 mL sample was extracted through the solid phase extraction (SPE) approach, utilizing Oasis HLB 6 cc (6 mL, 200 mg, waters) cartridges. Initially, the sample was acidified by adding HCl to obtain pH 2 and then spiked with 100 µL of 1000 ng mL^−1^ isotope-labeled internal standards. The sample was loaded into the Oasis HLB 6 cc (6 mL, 200 mg, waters) cartridges, conditioned sequentially with 6 ml methanol, 6 ml ultrapure water, and 6 ml acidified ultrapure water (pH = 2). After loading the spiked sample, interfering materials were washed with 20 mL ultrapure water. The cartridges were then dried under vacuum (at 5 bar pressure) for 5 min. Target analytes were eluted using 3 × 4 mL aliquots of methanol, which was then evaporated using GeneVac (EZ-2 Plus series) at a heating temperature of 45 ^O^C and a pressure of ≤ 200 mbar. Finally, analytes were recovered with 900 µL water: methanol (50:50, v/v) and 100 µL of a of a 1000 ng mL^−1^ isotope-labeled internal standard mixture of sulfamethoxazole and caffeine. The mixture was vortexed for 1 min, transferred to injection amber glass vial, and analytes were immediately analyzed by UPLC-QTOF.

### Instrument analysis and quality control measures

#### Instrument analysis

Target pharmaceuticals and their metabolites were quantified by the Waters ® Synapt G2 high-definition mass spectrometry (HDMS) system (Waters Inc., Milford, MA, USA). The apparatus was equipped with the Waters Acquity Ultra Performance Liquid Chromatography (UPLC) system connected to a quadrupole time-of-flight (QTOF) instrument. The instrument was equipped with A Kinetex® 1.7 µm EVO C18 100 Å column (100 mm length × 2.1 mm ID) for chromatographic separation of the target compounds. The column flow rate was set at 0.3 mL min^−1^ for the entire run, giving a total run time of 20 min, and the temperature was kept constant at 50 °C. Separation was performed using a reverse-phase gradient elution scheme from 97% H_2_O (Romil-UpS™, Microsep, South Africa) (with 0.1% formic acid (99 + % purity, Thermo Scientific, South Africa)) to 100% methanol (Romil-UpS™, Microsep, South Africa) (with 0.1% formic acid). The injection volume was 5 µl. Data acquisition and processing were done using the MassLynx™ (version 4.1) software (Waters Inc., Milford, MA, USA). Additional instrument parameters and chromatographic separation details are summarized in Supplementary Table S2.

#### Quality control measures

Six standard solutions ranging from 1 to 5000 ng mL^−1^ were used to obtain calibration curves for the targeted compounds (Supplementary Table S3). The calibration curves for sulfamethoxazole and caffeine were constructed using the concentration (x-axis) vs response ratio (y-axis) (i.e., area of analyte/area of internal standard). For the other analytes, their calibration curves were constructed using the concentration (x-axis) vs response (y-axis) (i.e., Area of the analyte). The final concentrations of all target compounds including sulfamethoxazole and caffeine were not corrected based on the recoveries of the internal standards. However, the isotope-labeled internal standards for sulfamethoxazole and caffeine were added to the extracts to facilitate the quantification of the response ratio (i.e., area of analyte/area of internal standard) for the two compounds primarily because their quantification is based on an internal standard calibration approach. The method validation results demonstrated acceptable recovery and precision for most analytes without the need for correction using internal standard. For example, Ac-SMX had recoveries above 90% in the soil and water samples (Supplementary Table S4 and Table S5)—which clearly shows that the internal standards were not necessary for this method. Furthermore, the % relative standard deviation and recoveries for the analytes quantified using the internal standard calibration approach—sulfamethoxazole and caffeine—compares well with the analytes quantified using the external standard calibration approach (Supplementary Table S4 and Table S5), showing that the internal standards were not needed for this method. The correlation coefficients (R^2^) of the calibration curves were greater than 0.9 (Supplementary Table S3). At least one method blank was analyzed with every batch of samples to determine the availability of the target compounds in the blanks. None of the target compounds were detected in the method blanks. In addition, ultra-LC MS grade methanol, which was used as a mobile phase, was injected after every 10 samples to check cross contamination. The limits of quantification (LOQs) and limits of detection (LODs) were determined from the calibration curves as concentrations that give a signal-to-noise (S/N) ratio of 10 and 3, respectively (Wooding, 2017). The LODs, LOQs, and the percentage recoveries of the target compounds in the soil and water are given in Supplementary Table S4 and Table S5, respectively.

### Statistical data analysis

A standard statistical analysis, including frequency distribution, mean, range, and standard deviation, was conducted to determine the occurrence and concentration levels of targeted pharmaceuticals in soil and groundwater. A principal component analysis (PCA) was conducted to explore potential relationships between pharmaceuticals in soils and groundwater; between soil physicochemical properties and pharmaceuticals in soils and groundwater; and farm management practices and pharmaceuticals in soils and groundwater, a principal component analysis (PCA) was conducted.

### Human health risk assessment

In South Africa, groundwater is mostly utilized in rural areas where treatment technologies are not available. Hence, there is a potential risk of pharmaceuticals to human health via drinking contaminated water. In this study, the human health risk assessment of pharmaceuticals detected in groundwater was performed for children– individuals younger than 12 years of age and adults– individuals older than 21 years of age. The risk to human health was assessed using the risk quotient (RQ) ratio, which is the relationship of the maximum measured concentration in drinking water with the amount of water that an individual can consume over a certain period. The calculation of the human health risk assessment involved the following three steps:

The first step is the calculation of the acceptable daily intake (ADI) of each pharmaceutical detected in groundwater according to the following equation proposed by Prosser and Sibley (2015) (Eq. 1).

1 $$\mathrm{ADI}=\frac{\mathrm{MTD}}{\mathrm{BW}\;\mathrm X\;\mathrm{SF}}$$

where ADI is the acceptable daily intake (µg kg^−1^ day^−1^), MTD is the minimum therapeutic dose (µg day^−1^), BW is the body weight for children and adults, taken as 30 and 70 kg, respectively (Sengar & Vijayanandan, 2022), and SF is the safety factor and was considered to be 1000.

The second step involves converting the ADI values of pharmaceuticals into the drinking water equivalent levels (DWEL). This is to ensure that the ADI takes into account the amount of water that an individual consumes daily and the number of days of exposure. The DWEL is given by the following equation proposed by Sharma et al. (2019) (Eq. 2).

2 $$\mathrm{DWEL}=\frac{\mathrm{ADI}\;\mathrm X\;\mathrm{BW}\;\mathrm X\;\mathrm{HQ}}{\mathrm{WI}\;\mathrm X\;\mathrm{FOE}}$$

where DWEL is the drinking water equivalent level (µg L^−1^), ADI is acceptable daily intake (µg kg^−1^ day^−1^), BW is the body weight for children and adults, HQ is the hazard quotient assumed as 1 (Sengar & Vijayanandan, 2022), WI is the water intake frequency (L day^−1^), and FOE is the frequency of water exposure taken as 365 days.

Finally, the last step involves the calculation of the risk quotient (RQ) ratio according to Sharma et al. (2019) (Eq. 3):

3 $$\mathrm{RQ}=\frac{\mathrm{MEC}}{\mathrm{DWEL}}$$

where RQ is the risk quotient ratio, DWEL is the drinking water equivalent level (µg L^−1^), and MEC is the maximum measured environmental concentration (µg L^−1^) in groundwater. RQ values less or equal to 0.2 indicate no appreciable risk, 0.2 to 1 indicates a need for well-refined risk assessment, and more than 1 indicates an appreciable risk to human health.

## Results and discussions

### Occurrence and concentration levels of targeted pharmaceuticals in representative cropland soils in Gauteng Province, South Africa

#### Occurrence of pharmaceuticals in the soil

**Fig. 2:**
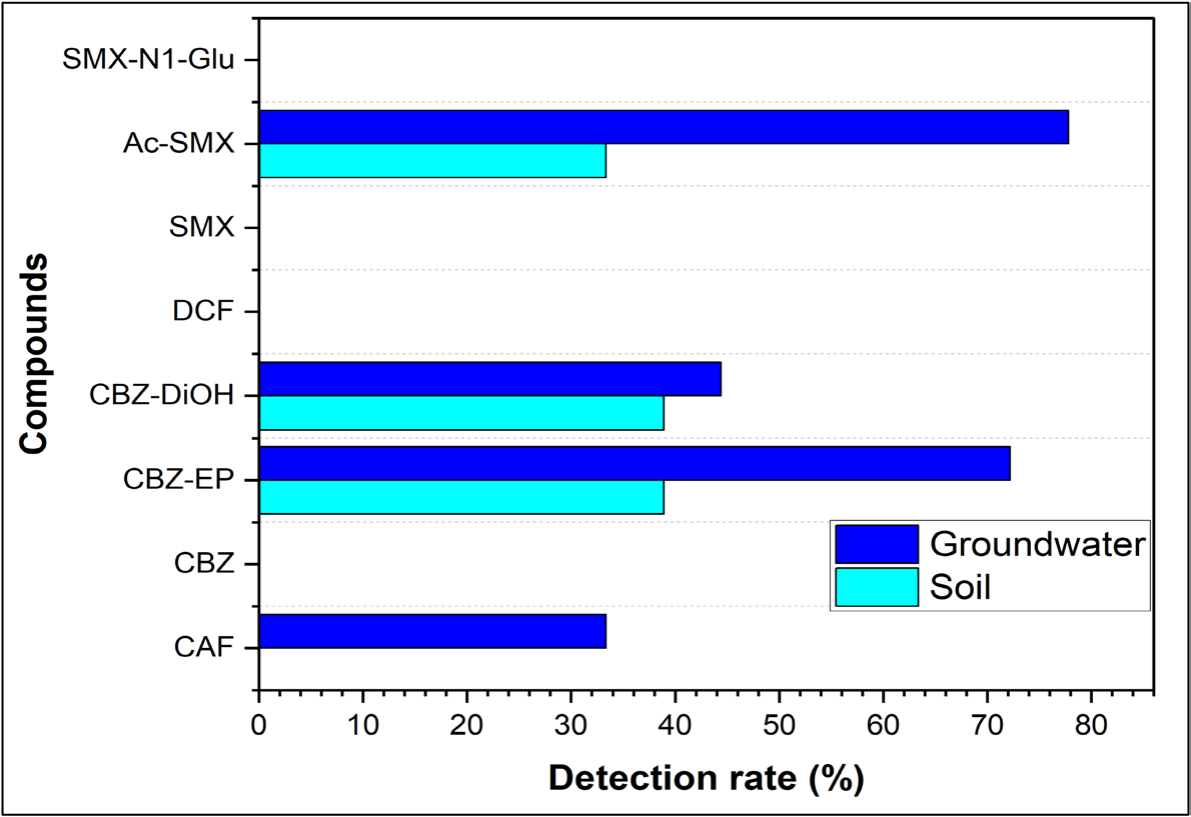
Detection rates of target pharmaceuticals in representative cropland soils and their underlying groundwater in Gauteng province, South Africa. *CAF* means caffeine, *CBZ* means carbamazepine, *CBZ-EP* means carbamazepine-10,11-epoxide, *CBZ-DiOH* means carbamazepine diol, *DCF* means diclofenac, *SMX* means sulfamethoxazole, *Ac-SMX* means N4-acetylsulfamethoxazole, and *SMX-N1-Glu* means sulfamethoxazole-N1-glucuronide

Three of the eight candidate pharmaceuticals – CBZ-EP, CBZ-DiOH, and Ac-SMX were detected in cropland soils of the study sites (Fig. 2). The remaining five pharmaceuticals were not detected in any of the soil samples (Fig. 2). Additionally, none of the eight targeted pharmaceuticals were detected in the reference soils (uncultivated natural grasslands) collected near the cropland areas, indicating that soil amendments were likely responsible for the presence of the three detected pharmaceuticals in the cropland soils. The detection rates of these three pharmaceuticals—CBZ-EP (33.3%), CBZ-DiOH (33.3%), and Ac-SMX (28.6%) were all below 50%, suggesting limited availability in the soil amendments and/or more rapid degradation following their accumulation. The detection rates of Ac-SMX observed in this study are significantly lower than those reported in cropland soils from Spain (47%) (García-Galán et al., 2013) and China (100%) (Liu et al., 2020). The high detection rate of Ac-SMX reported by Liu et al. (2020) and García-Galán et al. (2013) are primarily attributed to the use of municipal wastewater for irrigation, a practice that was not adopted by any of the farms examined in this research. On the other hand, the detection rates of CBZ-EP and CBZ-DiOH observed in this study are significantly higher than those reported in cropland soils from Tunisia (0%) (Fenet et al., 2012). The absence of both CBZ-EP and CBZ-DiOH in cropland soils from Tunisia can be explained by their observed high mobility and leaching in the soil due to poor adsorption caused by the low clay content (< 0.1%) (Fenet et al., 2012). In this study, cropland soils had much higher clay content ranging between 8 and 30% (Table 1), which might have enhanced the adsorption of the two compounds, thereby increasing their retention in the soil. Enhanced adsorption of both CBZ-EP and CBZ-DiOH in the soil due to an increase in clay content was also documented in other previous studies (Malvar et al., 2020).

#### Concentration levels of pharmaceuticals in the soil

The concentrations of CBZ-EP, CBZ-DiOH, and Ac-SMX ranged from not detected (n.d.) to 10.0 ng g^−1^ dry weight, n.d. to 353.5 ng g^−1^ dry weight, and n.d. to 59.1 ng g^−1^ dry weight, respectively (Table 2). The concentration of CBZ-EP found in this study is significantly higher than the ranges reported in cropland soils from Israel (n.d. to 0.8 ng g^−1^) (Paz et al., 2016) and Saudi Arabia (n.d.) (Picó et al., 2019). The absence of CBZ-EP in Saudi Arabian cropland soils can be attributed to its non-detection in municipal wastewater used for irrigation (Picó et al., 2019). Similarly, the low concentrations of CBZ-EP found in Israeli cropland soils may result from its absence in irrigation wastewater and potential transformation of the parent compound (Paz et al., 2016). The concentration of Ac-SMX observed in this study is considerably higher than that reported in cropland soils from Spain (n.d. to 1.38 ng g^−1^) (García-Galán et al., 2013) and China (10.9 to 23.29 ng g^−1^) (Liu et al., 2020). The low concentration of Ac-SMX in Chinese cropland soils can be explained by its introduction through wastewater, which has a lower concentration entering the soil during irrigation (ranging from 14.3 to 31.4 ng L^−1^, approximately 0.0143 to 0.0314 ng g^−1^) (Liu et al., 2020). Research has shown that when pharmaceuticals are introduced into soil via wastewater, they are more readily available for plant uptake and can easily leach into groundwater (Borgman & Chefetz, 2013; Gworek et al., 2021; Mordechay et al., 2018; Wu et al., 2010). This is because they are already in their solution form (Gworek et al., 2021; Mordechay et al., 2018). As previously mentioned, this practice was not adopted by any of the farms examined in this research.

**Table 2:** Concentration levels of the target pharmaceuticals in cropland soils and their underlying groundwater in Gauteng province, South Africa

	Soil [ng g^−1^ dry weight]			Groundwater [ng L^−1^]		
Compounds	Mean ± SD	Min	Max	Mean ± SD	Min	Max
CAF	n.d	n.d	n.d	8.95 ± 3.2	n.d	67.1
CBZ	n.d	n.d	n.d	n.d	n.d	n.d
CBZ-EP	2.51 ± 3.8	n.d	10.0	16.05 ± 13.2	n.d	106.7
CBZ-DiOH	52.42 ± 108.2	n.d	353.5	94.8 ± 29.5	n.d	506.7
DCF	n.d	n.d	n.d	n.d	n.d	n.d
SMX	n.d	n.d	n.d	n.d	n.d	n.d
Ac-SMX	9.23 ± 19.6	n.d	59.1	43.3 ± 65.3	n.d	113.8
SMX-N1-Glu	n.d	n.d	n.d	n.d	n.d	n.d

*n.d* means not detected, *CAF* means caffeine, *CBZ* means carbamazepine, *CBZ-EP* means carbamazepine-10,11-epoxide, *CBZ-DiOH* means carbamazepine diol, *DCF* means diclofenac, *SMX* means sulfamethoxazole, *Ac-SMX* means N4-acetylsulfamethoxazole, *SMX-N1-Glu* means sulfamethoxazole-N1-glucuronide, *SD* means standard deviation, *Min* means minimum concentration, and *Max* means maximum concentration

### Correlation between soil physicochemical parameters and soil pharmaceutical content

**Fig. 3:**
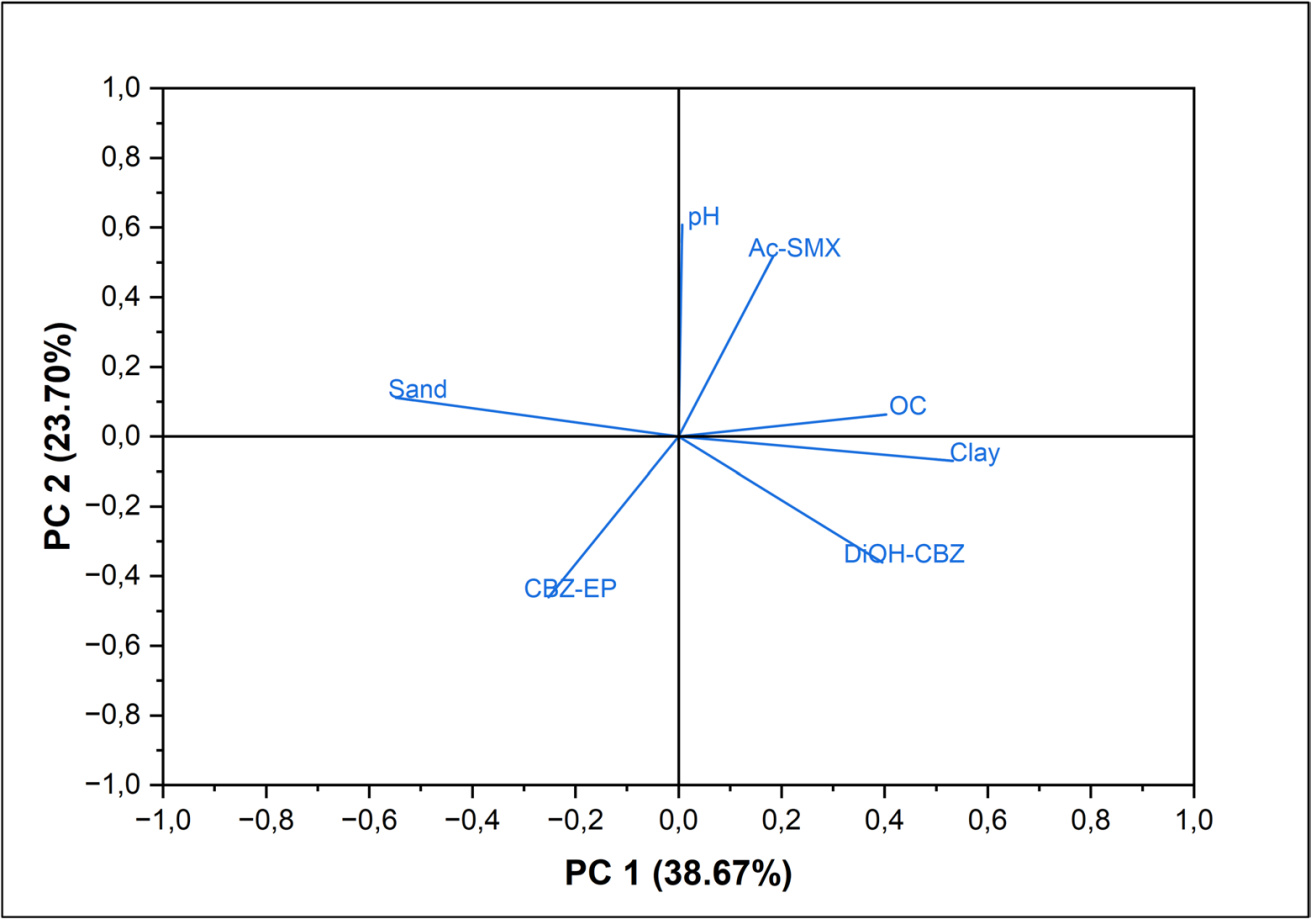
Loading plot showing correlations between physicochemical parameters and pharmaceuticals content in the soil. *CBZ-EP* means carbamazepine-10,11-epoxide, *CBZ-DiOH* means carbamazepine diol, and *Ac-SMX* means N4-acetylsulfamethoxazole

Previous studies have established correlations between soil physicochemical parameters and the content of pharmaceuticals in the soil (Chen & Akhtar, 2022; Gworek et al., 2021). Specifically, higher levels of soil organic matter and clay are associated with increased pharmaceuticals content in the soil (Gworek et al., 2021). This correlation can be attributed to the fact that an increase in soil organic matter and clay enhances the retention of pharmaceuticals in the soil by improving their adsorption on soil colloids (Gworek et al., 2021). In the current study, both clay and organic matter content displayed a strong positive correlation with both Ac-SMX and CBZ-DiOH content in the soil (Fig. 3). The results from this study also revealed a strong positive correlation between the soil pH and Ac-SMX content in the soil (Fig. 3). According to Gao and Pedersen (2005), the soil pH greatly influences the adsorption of sulfonamide antibiotics (Gao & Pedersen, 2005). When the pH exceeds the acid dissociation constants, sulfonamide antibiotics primarily exist in their negatively charged ion (anion), characterized by high-water solubility, high mobility, and poor adsorption (Gao & Pedersen, 2005). In the current study, over 70% of the cropland soils had a pH below the acid dissociation constant of Ac-SMX (pKa = 5.6). Hence, it is possible that Ac-SMX predominantly existed in their positively charged ions (cation), which is characterized by low water solubility, low mobility, and high adsorption (Gao & Pedersen, 2005).

### Occurrence and concentration levels of the targeted pharmaceuticals in groundwater underlying representative cropland soils

#### Occurrence of pharmaceuticals in the groundwater

Four out of the eight candidate pharmaceuticals – CBZ-EP, CBZ-DiOH, CAF, and Ac-SMX were detected in the groundwater beneath the cropland soils at the study sites (Fig. 2). The other four pharmaceuticals were not detected in any of the groundwater samples (Fig. 2). Among the detected candidate pharmaceuticals, three—CBZ-EP, CBZ-DiOH, and Ac-SMX—were also detected in the overlying soil samples (Fig. 2), suggesting a correlation between pharmaceutical contamination in soil and groundwater. While CAF was not detected in any overlying soil samples, it was present in the groundwater (Fig. 2). This can be attributed to its high-water solubility (21.7 g L^−1^ at 25 ^O^C) and low adsorbability (Log K_ow_ = 0.12) (Supplementary Table 2), which contribute to its increased mobility in soil and leachability into groundwater compared to the other candidate pharmaceuticals. The detection rates of Ac-SMX (77.8%) and CBZ-EP (72.3%) surpassed 50%, whereas CAF (34.1%) and CBZ-DiOH (43.4%) were within that threshold (Fig. 2).

The detection rate of CAF observed in this study is comparable to those reported for groundwater beneath cropland soils in the United States (36.6%) (Karnjanapiboonwong et al., 2011), yet it is significantly lower than the rates documented in Korea (88%) (Lee et al., 2019), Tunisia (100%) (Khezami et al., 2024), Taiwan (75%) (Lin et al., 2015), and China (67%) (Ma et al., 2018). Conversely, the detection rate of CAF in this study is higher than that reported for groundwater beneath cropland soils in Tokyo (16%) (Kuroda et al., 2012). In terms of CBZ-EP and CBZ-DiOH, the detection rates found in this study are significantly lower than those reported for groundwater beneath cropland soils in Tunisia (100%) (Fenet et al., 2012). The high detection rates of both CBZ-EP and CBZ-DiOH in Tunisian groundwater can be attributed to their high mobility and leaching in the soil, resulting from poor adsorption linked to low clay content (< 0.1%) (Fenet et al., 2012). Notably, the cropland soils examined in the current study exhibit a significantly higher clay content (8–30%) compared to those studied by Fenet et al. (2012). This higher clay content may have enhanced the retention of both CBZ-EP and CBZ-DiOH in the soil by increasing their adsorption capacity.

#### Concentration levels of pharmaceuticals in the groundwater

The concentration of CAF, CBZ-EP, CBZ-DiOH, and Ac-SMX varied from not detected (n.d) to 67.1, n.d to 106.7, n.d to 506.7, and n.d to 113.8 ng L^−1^, respectively (Table 2). Notably, the metabolite of carbamazepine, CBZ-DiOH, exhibited the highest concentration in the overlying soil samples from the study sites (Table 2), indicating a correlation between the presence of pharmaceuticals in soil and groundwater. Among the substances analyzed, CAF had the lowest concentration in groundwater (Table 2). This disparity can be attributed to several factors, including its minimal introduction into the soil—resulting in it not being detected in soil samples and dilution by groundwater, given its significantly higher water solubility compared to the other candidate pharmaceuticals (Supplementary Table 2). The concentration of CAF observed in this study is substantially higher than the ranges reported in groundwater beneath cropland soils from Korea (15 ng L^−1^) (Lee et al., 2019), Tokyo (11 ng L^−1^) (Kuroda et al., 2012), and China (7.96 to 34.1 ng L^−1^) (Ma et al., 2018), yet lower than levels found in Taiwan (2.3 to 89.3 ng L^−1^) (Lin et al., 2015), the USA (n.d to 166 ng L^−1^) (Karnjanapiboonwong et al., 2011), and Tunisia (33 to 1961 ng L^−1^) (Khezami et al., 2024). Likewise, the concentration of CBZ-EP and CBZ-DiOH recorded in this study is comparatively higher than those found in groundwater beneath cropland soils from Tunisia, which reported n.d to 6.1 and n.d to 15.8 ng L^−1^, respectively (Fenet et al., 2012). The lower concentration of both CBZ-EP and CBZ-DiOH in Tunisia can be explained by their minimal introduction into the soil, leading to their non-detection in soil samples, and/or more rapid degradation in soils characterized by poor adsorbability, as discussed earlier (Fenet et al., 2012).

### Correlation between water physicochemical parameters and water pharmaceutical content

**Fig. 4:**
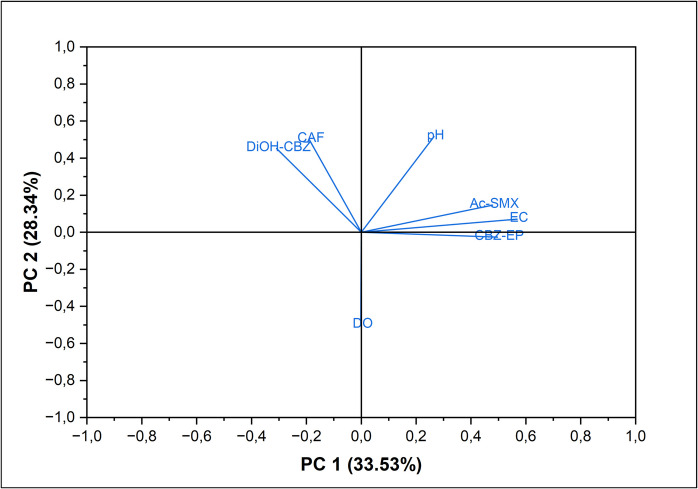
Loading plot showing correlations between physicochemical parameters and pharmaceuticals content in groundwater. *CAF* means caffeine, *CBZ-EP* means carbamazepine-10,11-epoxide, *CBZ-DiOH* means carbamazepine diol, and *Ac-SMX* means N4-acetyl sulfamethoxazole

Previous studies have established correlations between physicochemical parameters and the content of pharmaceuticals content in water (Zainab et al., 2021). According to Ohoro et al. (2022), there is a positive correlation between electrical conductivity and pharmaceutical content in water. Similarly, Khan et al. (2022) identified a positive correlation between pharmaceutical concentration and total dissolved solids. However, the same study found a negative correlation between water pH and pharmaceutical content (Khan et al., 2022). In this study, electrical conductivity exhibited a significant positive correlation with both CBZ-EP and Ac-SMX (Fig. 4). Notably, dissolved oxygen and pH showed no significant association with the candidate pharmaceuticals (Fig. 4).

### Correlation between farm management practices and the occurrence of pharmaceuticals in groundwater and cropland soils

#### Soil

**Fig. 5:**
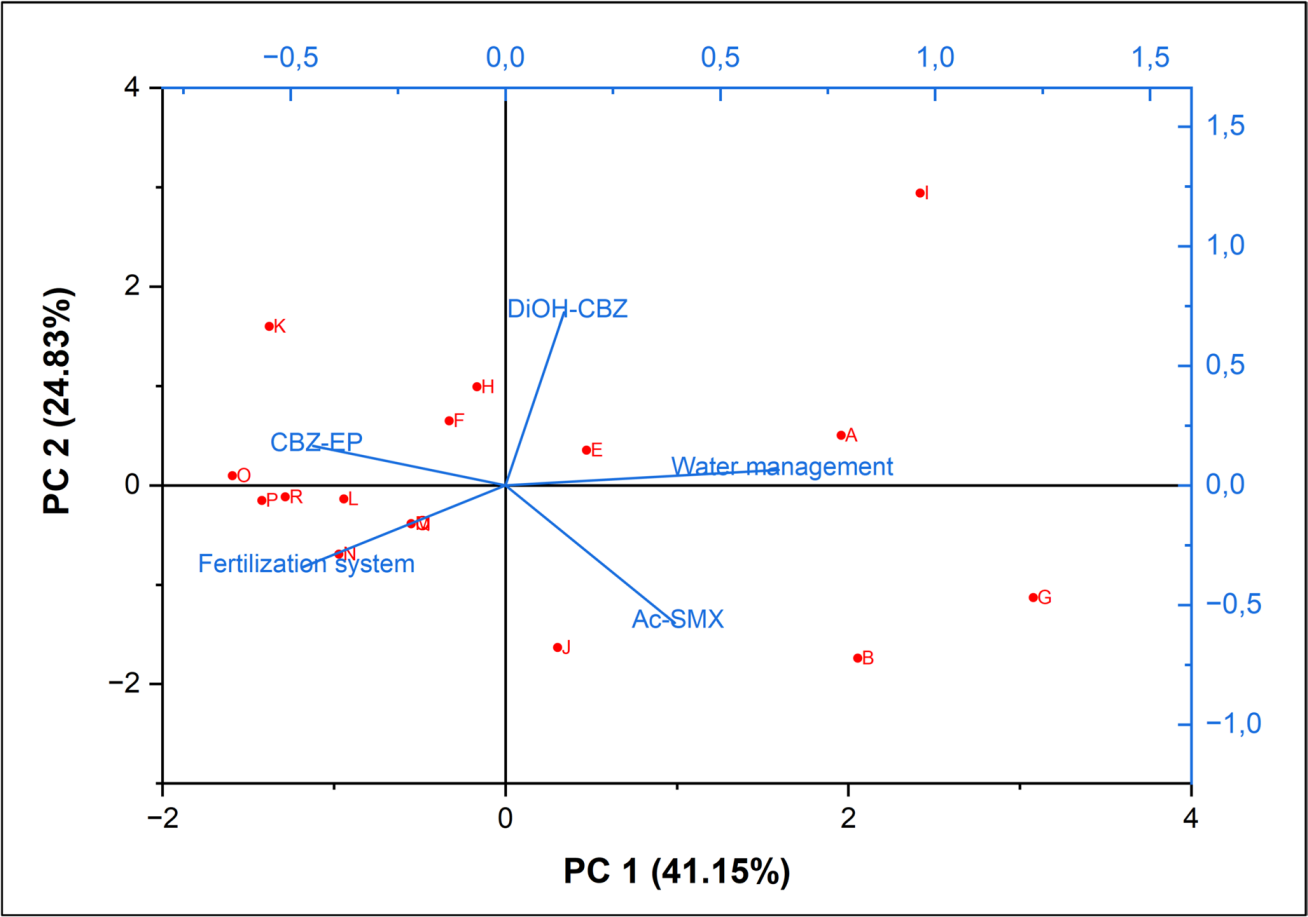
Bi-plot showing the correlation between the farm management practices and the occurrence of pharmaceuticals in the cropland soils in Gauteng province, South Africa. *CBZ-EP* means carbamazepine-10,11-epoxide, *CBZ-DiOH* means carbamazepine diol, and *Ac-SMX* means N4-acetyl sulfamethoxazole

Figure 5 illustrates the correlations between various farm management practices and the occurrence of pharmaceuticals in cropland soils. Notably, water management exhibited a strong negative correlation with the occurrence of CBZ-EP in the soil, indicating that increased water application likely reduces the occurrence of CBZ-EP. This effect may be attributed to dilution within the soil and potential leaching into the subsurface as water is added (Fenet et al., 2012). Conversely, the fertilization system demonstrated a positive correlation with CBZ-EP levels in the soil (Fig. 5), suggesting that the use of animal manures and/or commercial inorganic fertilizers may be responsible for its presence. This correlation is particularly evident in farms C, D, L, M, N, O, P, and R (Fig. 5), all of which utilize commercial inorganic fertilizers (Table 1), indicating these fertilizers are likely sources of CBZ-EP in the soil. Analysis of the commercial inorganic fertilizers used at the study sites revealed that CBZ-EP was present at levels above the limit of quantification (Supplementary Fig. 1). Furthermore, the results showed a strong positive correlation between water management and the occurrence of Ac-SMX and CBZ-DiOH in the soil (Fig. 5), suggesting that rainfall and/or irrigation water could be contributing to the presence of these two compounds. This correlation is closely linked to farms A, B, E, and J (Fig. 5), which predominantly rely on supplemental irrigation (Table 1), reinforcing the notion that irrigation water may be responsible for the detected levels of these compounds. There are no previous studies establishing the correlation between farm management practices and the occurrence of the candidate detected pharmaceuticals in the cropland soils, hence comparison of the correlation is not possible.

#### Groundwater

**Fig. 6:**
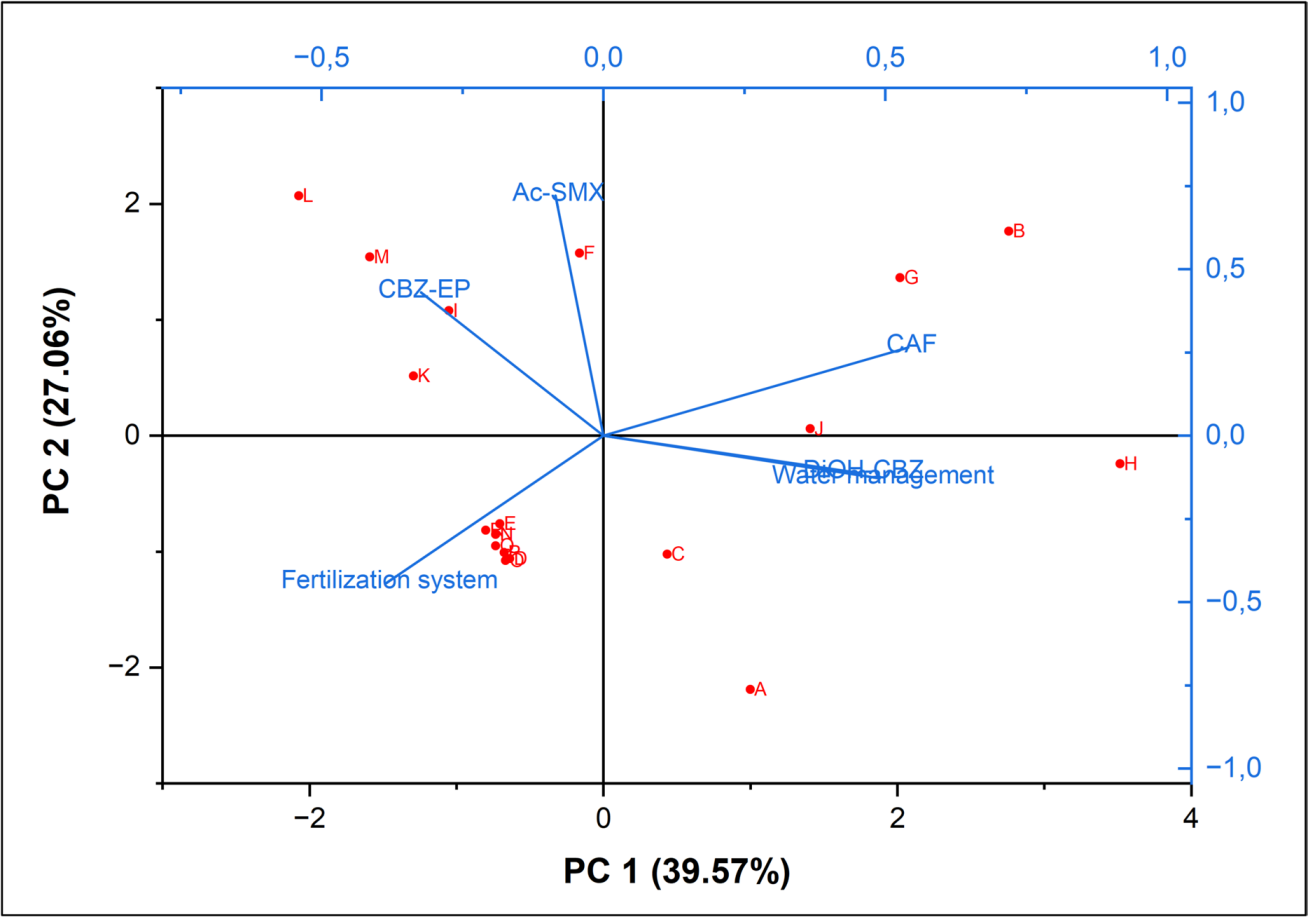
Bi-plot showing the correlation between the farm management practices and the occurrence of pharmaceuticals in groundwater underlying representative cropland soils in Gauteng province, South Africa. *CAF* means caffeine, *CBZ-EP* means carbamazepine-10,11-epoxide, *CBZ-DiOH* means carbamazepine diol, and *Ac-SMX* means N4-acetyl sulfamethoxazole

Figure 6 illustrates the correlations between farm management practices and the presence of pharmaceuticals in groundwater beneath cropland soils. Notably, water management exhibited a strong positive correlation with the occurrence of both CAF and CBZ-DiOH in groundwater (Fig. 6). This suggests that rainfall and/or irrigation water may have contributed to the detection of these two compounds. This correlation is particularly evident in farms H and J (Fig. 6), both of which utilized supplemental irrigation water (Table 1), further indicating that irrigation practices are likely a significant factor in the presence of these compounds in groundwater. Notably, there are no prior studies that have established a correlation between farm management practices and the occurrence of detected pharmaceuticals in the groundwater beneath cropland soils.

### Human health risk

In general, adults face a greater risk of being negatively impacted by the detected concentrations of pharmaceuticals in groundwater than children. This heightened risk is attributed to the higher daily water intake of adults, which averages 2 L day^−1^, compared to 1 L day^−1^ for children (Cunningham et al., 2010). The risk quotients (RQs) for CAF were 0.06 for children and 0.12 for adults, suggesting no significant risk to either children’s or adults’ health through drinking water exposure. These findings align with similar research conducted on groundwater in India (Sharma et al., 2019). Human health risk assessment for CBZ-EP, CBZ-DiOH, and Ac-SMX was not conducted due to the absence of established acceptable daily intake values for these compounds. These substances are metabolites formed endogenously in the human body and are not directly ingested orally, which limits the availability of relevant toxicological reference data.

## Conclusion

Four of the eight candidate pharmaceuticals – CAF, CBZ-EP, CBZ-DiOH, and Ac-SMX were detected in the groundwater beneath representative cropland soils in Gauteng Province, South Africa. Of these, three compounds, excluding CAF, were also found in the overlying soil samples, indicating a correlation between pharmaceutical contamination in soil and groundwater. Physicochemical parameters of both soil and water influenced the concentration of the targeted pharmaceuticals. Soil organic matter and clay content showed a strong positive correlation with the levels of Ac-SMX and CBZ-DiOH, suggesting that higher organic matter and clay content enhance the adsorption of these compounds in the soil. Additionally, electrical conductivity in water displayed a strong positive correlation with the concentrations of CBZ-EP and Ac-SMX. Farm management practices, including fertilization systems and water management, also impacted the occurrence of pharmaceuticals in both the cropland soil and groundwater. Specifically, the presence of CBZ-EP in soil and groundwater was positively correlated with the fertilization system, suggesting that fertilizers likely contributed to its presence. Conversely, the occurrence of CBZ-EP and Ac-SMX in both soil and groundwater was positively correlated with water management practices, indicating that water application may have facilitated the presence of these compounds. Future research incorporating multiple sampling periods would offer valuable insights into the effects of seasonal variations on groundwater levels and the transport of pharmaceuticals—an aspect that lies beyond the scope of the present study. Risk quotients showed that caffeine poses negligible risk to human health through drinking water exposure. However, future studies should focus on other metabolites of pharmaceuticals due to their higher concentrations in the soil and groundwater. This study highlights the importance of establishing the occurrence and baseline concentration levels of pharmaceuticals and their metabolites in agricultural settings and the underlying groundwater. Understanding these factors is essential to identifying the sources of pharmaceutical pollution and the conditions responsible for their persistence. Therefore, fertilizer guidelines should incorporate requirements for pharmaceutical levels to protect soil and groundwater resources.

## Supplementary Information

Below is the link to the electronic supplementary material.

Supplementary file1 (DOCX 113 KB)

## Data Availability

Data available upon reasonable request from the corresponding author.
