# Supplementary material for: Occurrence, concentration, and risk assessment of selected pharmaceuticals in representative cropland soils and their underlying groundwater in Gauteng province, South Africa
Source: Environ Monit Assess. 2025 Aug 6;197(9):986. doi: 10.1007/s10661-025-14436-1 (PMC12328511; doi:10.1007/s10661-025-14436-1)
Supplement: Supplementary file 1 — Supplementary file1 (DOCX 113 KB) [file 10661_2025_14436_MOESM1_ESM.docx]

**Occurrence, concentration, and risk assessment of selected pharmaceuticals in representative cropland soils and their underlying groundwater in Gauteng province, South Africa**

Matome Peter Ngoetjana ^a^, Eyob Habte Tesfamariam ^a*^, Sally Brown ^b^, Madelien Wooding ^c^, Matthys Dippenaar ^d^

^a^ University of Pretoria, Department of Plant and Soil Science, Pretoria, South Africa

^b^ University of Washington, School of Forest Resources, Seattle, USA

^c^ University of Pretoria, Department of Chemistry, Pretoria, South Africa

^d^ University of Pretoria, Department of Geology, Pretoria, South Africa

*Corresponding author

M.P. Ngoetjana

E-mail address: ngoetjanamp@gmail.com

ORCID: 0000-0003-4554-7037

E.H. Tesfamariam

E-mail address: eyob.tesfamariam@up.ac.za

ORCID: 0000-0003-0724-9864

S. Brown

E-mail address: slb@uw.edu

ORCID: 0000-0002-9405-571X

M. Wooding

E-mail address: madelien.wooding@up.ac.za

ORCID: 0000-0003-3869-7467

M. Dippenaar

E-mail address: matthys.dippenaar@up.ac.za

ORCID: 0000-0002-6807-1353

Supplementary Information:

**Table S1** Physicochemical parameters of the target pharmaceuticals (Extracted from: https://go.drugbank.com/)

| **Contaminant** | **Molar mass (g mol^-1^)** | **Water solubility (g L^-1^)** | **Log K_ow_** | **pKa** |
| --- | --- | --- | --- | --- |
| CBZ | 236.27 | 0.02 | 2.77 | 13.9 |
| CBZ-EP | 252.27 | 0.08 | 1.97 | 15.9 |
| CBZ-DiOH | 270.28 | 0.1 | 2.00 | 12.8 |
| SMX | 253.28 | 0.61 | 0.89 | 5.6 |
| Ac-SMX | 295.31 | 0.3 | 1.20 | 5.9 |
| SMX-N1-Glu | 429.4 | 2.55 | 0.22 | 3.57 |
| CAF | 194.19 | 21.7 | 0.12 | 14.0 |

*CAF* means caffeine, *CBZ* means carbamazepine, *CBZ-EP* means carbamazepine-10,11-epoxide, *CBZ-DiOH* means carbamazepine diol, *DCF* means diclofenac, *SMX* means sulfamethoxazole, *Ac-SMX* means N4-acetylsulfamethoxazole, and *SMX-N1-Glu* means sulfamethoxazole-N1-glucuronide.

**Table S2** Instrument parameters

| **Compound** | **Ionization mode** | **Retention time (min)** | **Quantification ion (m/z)** |
| --- | --- | --- | --- |
| CAF | Positive | 4.8 | 195.0880 |
| CBZ | Positive | 8.8 | 237.1035 |
| CBZ-EP | Positive | 7.5 | 253.0971 |
| CBZ-DiOH | Positive | 4.9 | 271.1096 |
| DCF | Positive | 6.4 | 215.0710 |
| SMX | Positive | 5.3 | 254.0595 |
| Ac-SMX | Positive | 6.3 | 296.0707 |
| SMX-N1-Glu | Positive | 4.4 | 254.0605 |

*CAF* means caffeine, *CBZ* means carbamazepine, *CBZ-EP* means carbamazepine-10,11-epoxide, *CBZ-DiOH* means carbamazepine diol, *DCF* means diclofenac, *SMX* means sulfamethoxazole, *Ac-SMX* means N4-acetylsulfamethoxazole, and *SMX-N1-Glu* means sulfamethoxazole-N1-glucuronide.

**Table S3** Linearity of the calibration curve

|  | **Linearity** | | | |
| --- | --- | --- | --- | --- |
| **Target compounds** | **Range (ng mL^-1^)** | **Calibration level** | **Regression equation** | **R^2^** |
| CAF | 1 – 5000 | 6 | y = 1.09231x + 0.0618 | 0.991 |
| CBZ | 1 – 5000 | 6 | y = 10592.7x - 69.5151 | 0.989 |
| DCF | 1 – 5000 | 6 | y = 3216.31x - 28.1044 | 0.991 |
| SMX | 1 – 5000 | 6 | y = 1.20676x - 0.0136747 | 0.993 |
| SMX-N1-Glu | 1 – 5000 | 6 | y = 1459.13x - 18.068 | 0.994 |
| Ac-SMX | 1 – 5000 | 6 | y = 25.9956x + 0.397317 | 0.977 |
| CBZ-EP | 1 – 5000 | 6 | y = 1049.79x - 7.25038 | 0.993 |
| CBZ-DiOH | 1 – 5000 | 6 | y = 109.529x - 0.736874 | 0.993 |

*CAF* means caffeine, *CBZ* means carbamazepine, *CBZ-EP* means carbamazepine-10,11-epoxide, *CBZ-DiOH* means carbamazepine diol, *DCF* means diclofenac, *SMX* means sulfamethoxazole, *Ac-SMX* means N4-acetylsulfamethoxazole, and *SMX-N1-Glu* means sulfamethoxazole-N1-glucuronide.

**Table S4** Limit of detections (LODs), limit of quantifications (LOQs), percentage recoveries (%), and relative standard deviation (RSD) for the soil samples (n=3)

| **Compound** | **LODs (ng g^-1^)** | **LOQs (ng g^-1^)** | **Recovery (%); RSD (%)** |
| --- | --- | --- | --- |
| CAF | 0.02 | 0.05 | 99.8; 15 ^a^ |
| CBZ | 0.62 | 2.10 | 115; 8 ^b^ |
| DCF | 6.40 | 21.00 | 60.5; 33 ^b^ |
| SMX | 1.00 | 3.90 | 115; 10 ^a^ |
| SMX-N1-Glu | 2.00 | 6.60 | 112; 18 ^b^ |
| Ac-SMX | 1.00 | 3.40 | 96.8; 5 ^b^ |
| CBZ-EP | 0.31 | 1.00 | 62;12 ^b^ |
| CBZ-DiOH | 3.10 | 10.00 | 70.1; 6 ^b^ |

^a^ Percentage recovery (%) from concentrations calculated from an internal standard calibration curve. ^b^ Percentage recovery (%) from concentrations calculated from an external calibration curve. *CAF* means caffeine, *CBZ* means carbamazepine, *CBZ-EP* means carbamazepine-10,11-epoxide, *CBZ-DiOH* means carbamazepine diol, *DCF* means diclofenac, *SMX* means sulfamethoxazole, *Ac-SMX* means N4-acetylsulfamethoxazole, and *SMX-N1-Glu* means sulfamethoxazole-N1-glucuronide.

**Table S5** Limit of detections (LODs), limit of quantifications (LOQs), percentage recoveries (%), and relative standard deviation (RSD) for water samples (n=3)

| **Compound** | **LODs (ng L^-1^)** | **LOQs (ng L^-1^)** | **Recovery (%); RSD (%)** |
| --- | --- | --- | --- |
| CAF | 0.03 | 0.10 | 100; 6 ^a^ |
| CBZ | 1.25 | 4.15 | 90.1; 12 |
| DCF | 12.85 | 42.83 | 95.4; 16 |
| SMX | 2.03 | 6.77 | 102; 10 ^a^ |
| SMX-N1-Glu | 3.99 | 13.30 | 111;12 |
| Ac-SMX | 2.04 | 6.81 | 95.9; 8 |
| CBZ-EP | 0.61 | 2.04 | 79.2; 5 |
| CBZ-DiOH | 6.16 | 20.53 | 80.1; 10 |

^a^ Percentage recovery (%) from concentrations calculated from an internal standard calibration curve. ^b^ Percentage recovery (%) from concentrations calculated from an external calibration curve *CAF* means caffeine, *CBZ* means carbamazepine, *CBZ-EP* means carbamazepine-10,11-epoxide, *CBZ-DiOH* means carbamazepine diol, *DCF* means diclofenac, *SMX* means sulfamethoxazole, *Ac-SMX* means N4-acetylsulfamethoxazole, and *SMX-N1-Glu* means sulfamethoxazole-N1-glucuronide.

**Fig. S1** Concentration levels of carbamazepine-10,11-epoxide in commercial inorganic fertilizers
